# The elevated levels of urinary angiotensinogen are correlated with the severity of idiopathic membranous nephropathy

**DOI:** 10.1186/s12882-018-1165-1

**Published:** 2018-12-12

**Authors:** Ziyong Tang, Yue Wang, Liyuan Tao, Yanhong Guo, Yimu Zheng, Danxia Zheng

**Affiliations:** 1 Department of Nephrology, Peking University Third Hospital, No. 49, North Garden Road, Haidian District, Beijing, 100191 China; 2 Department of Clinical Epidemiology, Peking University Third Hospital, Beijing, China

**Keywords:** Urinary angiotensinogen, Idiopathic membranous nephropathy, Disease severity

## Abstract

**Background:**

Immunosuppressive treatment will predispose an idiopathic membranous nephropathy (iMN) patient to opportunistic infections. Disease severity is one of the main concerns for making the treatment decision. Urinary angiotensinogen (UAGT) level has been shown highly correlated with intrarenal renin-angiotensin system (RAS) activity and severity of chronic kidney diseases (CKD). We aimed to test the relationship between the UAGT level and the severity of iMN.

**Methods:**

This cross-sectional study included a total of 48 biopsy-proven iMN patients, 46 minimal change disease (MCD) patients, and 44 healthy volunteers. The clinical and laboratory data and urine samples were collected from all subjects before the use of RAS inhibitors. We determined the UAGT levels with a method of enzyme-linked immunosorbent assay.

**Results:**

The UAGT levels were not different between the iMN (277.05 ± 61.25, μg/g.Cr) and MCD patients (244.19 ± 40.24, μg/g.Cr), but both of them were significantly higher than those of healthy controls (6.85 ± 1.10, μg/g.Cr). UAGT levels were correlated negatively with serum albumin (*r* = − 0.393, *p* = 0.006) and estimated glomerular filtration rate (eGFR) (*r* = − 0.352, *p* = 0.014) and positively with 24-h proteinuria (*r* = 0.614, *p* < 0.001) in iMN patients but not in MCD patients. Multivariate linear regression analysis revealed that only proteinuria independently determinate the levels of UAGT (β = 0.649, p < 0.001) in iMN patients.

**Conclusions:**

UAGT levels were correlated negatively with serum albumin and glomerular filtration rate and positively with proteinuria in iMN patients at the onset. This suggests that elevated levels of UAGT are associated with the severity of iMN. The UAGT level may be used as a cofactor for deciding immunosuppressive therapy in iMN patient.

## Background

Idiopathic membranous nephropathy (iMN) is a common cause of adult-onset nephrotic syndrome and increased by more than twice over the past decade in China [1]. Most iMN patients maintained renal function for prolonged periods and likely to have spontaneous remission (SR). Only about 30% of the iMN patients with nephrotic proteinuria will develop end stage renal disease (ESRD) without appropriate treatment [2]. Even with restrictive treatment, iMN patients with nephrotic proteinuria had quite favorable long-term outcomes. They had an overall renal survival of 86% after 10 years [3]. As immunosuppressive treatment will predispose an iMN patient to opportunistic infections and other severe side effects, the Kidney Disease Improving Global Outcomes (KDIGO) guidelines recommend using immunosuppressive agents only in patients at high risk for developing kidney failure [4].

It is of interest to many researchers to distinguish these high-risk iMN patients. Cattran group created the Toronto Risk Score which accurately predicted the progression of iMN and was based on the change in creatinine clearance and the maximum of proteinuria during a 6-month period [5]. Prolonged observation is necessary for this model, and the patients were kept in uncertainty during this period. Alternatively, several urinary markers have been proposed to predict progression in iMN [6, 7], especially low-molecular-weight proteins including α1 and β2 microglobulins [2].

The intrarenal renin-angiotensin system (RAS) activation has several functions not only regulating blood pressure but also developing renal cell growth, and affecting renal function eventually [8, 9]. Angiotensinogen (AGT) is the sole precursor of all angiotensin peptides. Human AGT has 485 amino acids and can be present in non-glycosylation or glycosylation forms in serum with a molecular weight of 53 kDa or 75 kDa respectively [10]. Serum AGT cannot be freely filtered through the glomerular basement membrane in normal conditions. Urinary AGT (UAGT) was mainly generated locally and accurately reflect intrarenal RAS activity [11, 12]. The correlations between elevated UAGT levels and the severity of kidney disease had been reported in immunoglobulin A nephropathy and diabetic nephropathy [13–16].

It had been shown that intrarenal RAS was activated in progressive iMN and elevated levels of in situ generation of angiotensin II and angiotensin-converting enzyme were associated with the disease severity [17]. However, the correlation between UAGT and disease severity of iMN had not been extensively studied. In the present study, we attempted to explore the relationship between UAGT and severity of iMN patients at the onset. We aimed to find another urine marker which will add to the distinguishing ability of the above-mentioned risk markers.

## Methods

### Study populations

This is a cross-sectional study. All the patients with iMN who attended our renal unit from April of 2011 to April of 2013 were recruited in this study. iMN was proven by kidney biopsy, according to the KDIGO clinical practice guidelines for glomerulonephritis [4]. Diagnostic features included capillary wall thickening, normal cellularity, IgG and C3 deposition along capillary walls upon immunofluorescence, and sub-epithelial deposits visible by electron microscopy. Patients with systemic diseases such as rheumatic diseases, malignant tumors, diabetes mellitus, infections with hepatitis B or C virus, and other diseases associated with secondary membranous nephropathy were excluded. The subjects with urinary tract infections and a history of renal transplantation were excluded. Those were also excluded if they took RAS inhibitors or were treated with immunosuppressive drugs at the time of sample collection. Biopsy-proven MCD patients and healthy volunteers with no evidence of kidney disease were enrolled as controls. The protocol of this study was approved by Peking University Third Hospital Medical Science Research Ethics Committee and informed consent was obtained before the initial collection of urine samples.

### Clinical and laboratory data

Data pertaining to baseline characteristics were collected from kidney biopsy registration sheets. For each patient, baseline data including age, gender, duration of symptoms before biopsy, height, body weight, blood pressure, proteinuria (24-h urinary protein excretion) and urine sediment examination were recorded. Blood and urine samples were collected in the early morning of the biopsy day. Urine samples were frozen at the temperature of − 80 °C for further evaluation. Serum creatinine was measured with a Jaffe alkaline picrate method and serum albumin was measured using a bromcresol green assay. All measurements were performed on a Beckman Coulter AU5800 chemistry analyzer in the clinical laboratory of Peking University Third Hospital [18]. Estimated glomerular filtration rate (eGFR) was calculated with the CKD-EPI creatinine equation recommended by KDIGO and expressed as ml/min/1.73 m^2^ of body surface area [19] .

### Measurement of urinary angiotensinogen

UAGT levels were measured with a commercially available sandwich enzyme-linked immunosorbent assay (ELISA) kit (IBL, Fujioka, Japan), which has intra-assay and inter-assay variation coefficients of 4.4 and 4.3%. Highly purified angiotensinogen from human plasma was used as the standard. The UAGT level was adjusted by urine creatinine concentration and expressed as μg/g.Cr.

### Statistical analysis

Continuous variables were expressed as means ± SD while categorical variables were expressed as numbers and percentages. The independent t-test was used for the comparison between two groups, and one-way ANOVA was used for three or more groups. The Bonferroni test was used for the Post Hoc multiple comparisons. The chi-square test was employed for comparison of categorical variables between groups. Pearson’s correlation analysis was performed to explore the possible relationship between continuous variables. The significant variables identified by Pearson’s correlation were further selected into a multiple linear regression model to determine whether they have independent effects. All tests were two-sided and *p* < 0.05 was considered significant. Statistical analysis was performed using SPSS software, version 23.0 (SPSS Inc., Chicago, IL, USA).

## Results

### Baseline characteristics and UAGT levels in iMN patients, MCD patients, and normal subjects

**Fig. 1:**
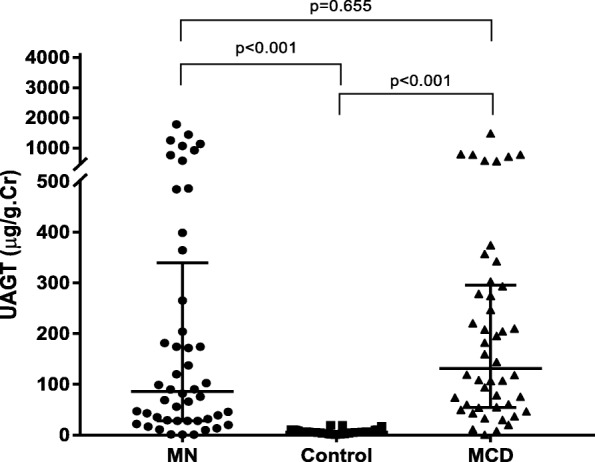
Comparison of the UAGT levels among the iMN patients, MCD patients and normal healthy subjects. Both of the iMN patients and MCD patients had higher UAGT levels than normal subjects; there were no differences between the iMN patients and MCD patients

Baseline characteristics were summarized in Table 1. A total of 48 iMN patients, 46 MCD patients, and 44 healthy volunteers were included in this study. The iMN patients were from 24 to 76 years old; the MCD patients were from 17 to 71 years old; and the healthy volunteers were from 28 to 57 years old. Profiles including gender and serum uric acid were not different among the three groups. The levels of blood pressure, eGFR, serum albumin and proteinuria were different among groups. Also, the UAGT levels were significantly different among the three groups. As was shown in Fig. 1, the iMN patients had similar levels of UAGT as MCD patients, while both of them had significantly higher UAGT levels than normal subjects (*p* < 0.001).

**Table 1:** Baseline clinical and laboratory data and UAGT levels of iMN patients, MCD patients, and normal subjects

	iMN (*n* = 48)	MCD (*n* = 46)	Normal (*n* = 44)	*P* value
Age (years)	53 ± 2	40 ± 2^##^	41 ± 2	< 0.001**
Sex (male/female)	33/15	25/21	24/20	0.350
BMI (kg/m^2^)	25.2 ± 0.5	23.0 ± 0.5	22.0 ± 0.6	0.010*
Systolic BP (mmHg)	130.0 ± 2.7	124.5 ± 2.4	120.3 ± 1.1	0.009**
Diastolic BP (mmHg)	80.1 ± 1.8	76.0 ± 1.7	71.9 ± 1.1	0.002**
Uric acid (μmol/L)	356.3 ± 11.0	351.4 ± 10.0	379.6 ± 3.3	0.067
Serum creatinine (μmol/L)	71.2 ± 2.6	65.4 ± 2.0	68.8 ± 2.0	0.041*
Serum albumin (g/L)	29.6 ± 1.0	21.8 ± 1.1^##^	42.4 ± 0.5	< 0.001**
eGFR (ml/min/1.73m^2^)	37.1–143.0 (100.0 ± 3.0)	78.0–146.8 (110.0 ± 2.4)	48.9–162.9 (114.6 ± 4.5)	0.008**
Proteinuria (g/d)	6.6 ± 0.7	8.5 ± 0.7	0.100 ± 0.004	< 0.001**
UAGT (μg/g.Cr)	277.1 ± 61.3	241.2 ± 42.4	6.5 ± 0.7	< 0.001**

*BMI* body mass index, *BP* blood pressure, *eGFR* estimated glomerular filtration rate, *UAGT* urinary angiotensinogen. *P < 0.05, **P < 0.01 among the three groups. ^##^*p* < 0.01 between iMN and MCD patients.

### Relationships between UAGT and other variables in the iMN or MCD patients

**Fig. 2:**
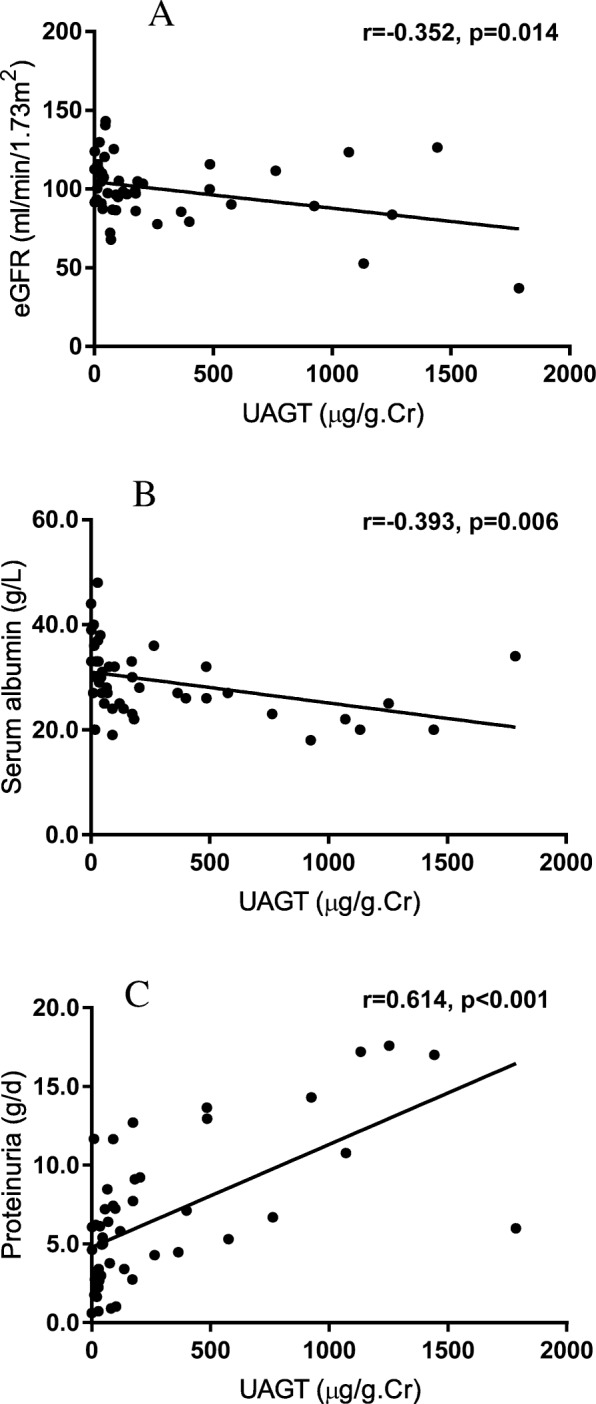
Correlations between UAGT and other variables in iMN patients: **A**), UAGT levels were negatively correlated with eGFR (*r* = -0.352, *p* = 0.014); **B**), UAGT levels were negatively correlated with serum albumin (*r*=-0.393, *p* = 0.006); **C**), UAGT levels were positively correlated with proteinuria (*r* = 0.614, *p* < 0.001).

Pearson’s correlations between UAGT levels and other parameters were performed in the iMN patients and MCD patients respectively and the results were shown in Table 2. In iMN patients, the UAGT levels were correlated negatively with serum creatinine (*p* = 0.033), serum albumin (*p* = 0.006) and eGFR (*p* = 0.014) and positively with systolic blood pressure (*p* = 0.045) and proteinuria (*p* < 0.001). However, none of the above parameters was found to be significantly correlated with UAGT in MCD patients. Linear regression analysis further confirmed that UAGT levels were linearly correlated with eGFR (*r* = − 0.352, p = 0.014), as well as with serum albumin (*r* = − 0.393, *p* = 0.006) and proteinuria (*r* = 0.614, p < 0.001). (See Fig. 2).

**Table 2:** Pearson’s correlation of UAGT and other variables in the iMN or MCD patients

	iMN		MCD	
Factors	Coefficient	*P* value	Coefficient	*P* value
Age (years)	0.193	0.188	−0.073	0.723
BMI	0.063	0.670	−0.008	0.971
SBP (mmHg)	0.291	0.045*	−0.186	0.362
DBP (mmHg)	0.133	0.367	−0.089	0.667
Uric acid (μmol/L)	−0.161	0.281	−0.133	0.583
Serum creatinine (μmol/L)	0.308	0.033*	0.253	0.212
Serum albumin (g/L)	−0.393	0.006**	0.051	0.804
eGFR (ml/min/1.73m^2^)	−0.352	0.014*	−0.186	0.362
Proteinuria (g/d)	0.614	< 0.001**	−0.229	0.261

*BMI* body mass index, *SBP* systolic blood pressure, *DBP* diastolic blood pressure, *eGFR* estimated glomerular filtration rate, *UAGT* urinary angiotensinogen. *P < 0.05, **P < 0.01.

### Multivariate analysis of the independent determinant for UAGT in iMN patients

When age, systolic blood pressure, serum albumin, eGFR, and proteinuria were included in a multivariate linear regression analysis model, only proteinuria was shown to be the independent determinant of UAGT in iMN patients (β = 0.590, *p* = 0.002). (See Table 3).

**Table 3:** Multivariate linear regression analysis between UAGT and other variables in iMN patients

Variables	Unstandardized/standardized β	*t*	*P* value
Age (years)	−3.751/− 0.134	− 0.790	0.434
SBP (mmHg)	−0.170/− 0.007	−0.055	0.957
Serum albumin (g/L)	2.187/0.034	0.203	0.840
eGFR (ml/min/1.73m^2^)	−6.356/0.303	−1.702	0.096
Proteinuria (g/d)	0.055/0.590	3.386	0.002**

*SBP* systolic blood pressure, *eGFR* estimated glomerular filtration rate. **P < 0.01.

## Discussion

In the present study, we found that UAGT levels were higher in glomerulonephritis patients than normal subjects; meanwhile, no differences of UAGT levels were found between iMN patients and MCD patients. Further analysis indicated that the UAGT levels were correlated with several parameters relevant to disease severity of iMN patients, including serum albumin, eGFR, and proteinuria. However, these correlations were found not statistically significant in MCD patients. Multivariate regression analysis showed that proteinuria was the independent determinant of UAGT levels in iMN patients.

Defining factors relating to the disease severity is crucial to iMN patients and of interest to many researchers. The natural clinical course of iMN is that one-third tend to have spontaneous remission, one-third have stable renal function, and one-third progress to renal failure. If treated properly, the iMN patients will have overall renal survival of about 86% after 10 years [3]. However, immunosuppressive treatments for iMN, including corticosteroids, alkylating agents, and calcineurin inhibitors, will predispose the patients to opportunistic infections. In addition, alkylating agents, such as cyclophosphamide, increased cancer risk about threefold [20]. As the renal survival of iMN is quite good and the immunosuppressive treatment is toxic, KDIGO guidelines recommend that immunosuppressive agents be used only in patients at high risk of developing renal failure [4]. Some authors suggested that conservative treatment may be extended beyond 6 months of follow-up [3]. However, the prolonged observation period required to identify a high-risk patient would expose the patient to severe complications of nephrotic syndromes, such as edema, thrombosis, and infections. Many researchers have attempted to identify early predictors for prognosis which would allow early treatment and rapid disappearance of nephrotic syndrome in high-risk patients and avoid unnecessary exposure to toxic therapy in low-risk patients. Dr. van den Brand JA et al. had shown that low-molecular-weight proteins including α1 and β2 microglobulins were associated with the disease severity and prognosis of iMN patients [2, 21]. We aimed to find another marker that was relevant to iMN severity.

In the present study, UAGT levels were shown to be associated with severity and predictive markers of iMN patients, including low serum albumin, low eGFR, and high proteinuria. These risk factors had been proven by several studies. Low serum albumin levels at the onset were found to be associated with poor renal prognosis of iMN patients by Huh et al. [22]. High level of serum creatinine at the diagnosis was shown to be the main predictor for progression to ESRD in membranous nephropathy patients [23]. Also, severe proteinuria was used as a major predictor for renal prognosis of iMN in a conventional predictive model, Toronto Risk Score [5]. Based on this, our data supported that the levels of UAGT were associated with the severity of iMN patients.

It is doubted whether the elevated levels of UAGT were nonspecific consequences of proteinuria as both iMN and MCD patients presented heavy proteinuria in our study. Our analysis further showed that UAGT was correlated with the above parameters only in iMN patients, but not in MCD patients. This difference supported the hypothesis that UAGT was not a non-specific consequence of proteinuria as both iMN and MCD patients had severe proteinuria. Actually, several studies supported this hypothesis. Kobori and coworkers found low UAGT levels in MCD patients [24]. Jang’s work showed that UAGT was predominantly composed of filtered AGT from the systemic circulation in patients with nephrotic-range proteinuria. However, this phenomenon only presented in MCD or diabetic nephropathy patients but not in iMN patient [25]. A few authors suggested that UAGT levels reflected the intrarenal RAS activity and severity of chronic kidney diseases rather than a non-specific consequence of proteinuria [14–16, 24, 26].

One of the interesting findings of the present study was that UAGT was significantly correlated with several parameters in iMN patients but not in MCD patients. We supposed that this may be due to several mechanisms. Firstly, as had been shown by Dr. Jang et al., the origin of UAGT was different in different subtypes of glomerulonephritis. UAGT was correlated with serum AGT in MCD patients but not in iMN patients. They suggested that UAGT was mainly filtered from the serum in nephrotic MCD but produced locally in iMN [25]. Secondly, the different pathogenesis of MCD and iMN may cause the different behavior of UAGT. The activation of the RAS system is likely consequence of the primary lesion in the kidney. The most recent finding for the pathogenesis of iMN suggested that it was a humoral autoimmune disease caused by circulating auto-antibodies against receptors on the podocyte, notably anti-phospholipase A2 receptor (PLA2R) [27]. However, MCD is dominantly believed to be caused by dysfunctions of T cells [28–30]. Furthermore, we noticed that, clinically, RAS inhibitors may reduce the activity of iMN but not MCD. The mechanisms why UAGT behaves differently in iMN and MCD need to be further elucidated.

A few limitations remained in our study. First, the number of iMN patients in our study is relatively small. As it is a pilot study to explore the utility of UAGT as a biomarker for the severity of iMN patients, we will expand the observation number inspired by these promising results. Second, the cross-sectional character of the present study cannot provide proof of the predictive ability of UAGT on kidney outcomes. We can only suggest that the UAGT levels were associated with the severity markers of iMN patients. A prospective cohort study is needed to test the predictive value of UAGT in iMN patients. Another limitation of our study is the lack of anti-PLA2R antibody which had been suggested highly correlated with the disease severity of iMN in recent years. This was due to the cross-sectional nature of the study and measurement of anti-PLA2R antibody was not practicable at that time. We are now measuring anti-PLA2R antibody titer of all iMN patients in our clinic.

## Conclusions

In summary, UAGT levels were significantly higher in iMN patients and MCD patients than those of normal healthy controls. In iMN patients, UAGT levels were significantly correlated negatively with serum albumin and eGFR, and positively with 24-h proteinuria. Proteinuria was found to be an independent determinant of UAGT. These correlations were found not statistically significant in MCD patients although they had similar levels of proteinuria as well. We supposed that elevated levels of UAGT were correlated with the severity parameters of iMN patients and may be used as a cofactor for deciding immunosuppressive therapy for iMN patient.

## Abbreviations

ANOVA: Analysis of variance
BMI: Body mass index
CKD: Chronic kidney disease
CKD-EPI: CKD Epidemiology Collaboration
eGFR: Estimated glomerular filtration rate
ELISA: Enzyme-linked immunosorbent assay
ESRD: End stage renal disease
iMN: Idiopathic membranous nephropathy
KDIGO: Kidney Disease Improving Global Outcomes
MCD: Minimal change disease
PLA2R: Phospholipase A2 receptor
RAS: Renin-angiotensin system
SD: Standard deviation
SR: Spontaneous remission
UAGT: Urinary angiotensinogen
