# Correction to: The elevated levels of urinary angiotensinogen are correlated with the severity of idiopathic membranous nephropathy

**DOI:** 10.1186/s12882-019-1229-x

**Published:** 2019-02-13

**Authors:** Ziyong Tang, Yue Wang, Liyuan Tao, Yanhong Guo, Yimu Zheng, Danxia Zheng

**Affiliations:** 1 Department of Nephrology, Peking University Third Hospital, No. 49, North Garden Road, Haidian District, Beijing, 100191 China; 2 Department of Clinical Epidemiology, Peking University Third Hospital, Beijing, China

**Correction to: BMC Nephrology (2018) 19**:**357**

**https://doi.org/10.1186/s12882-018-1165-1**

Following publication of the original article [1], it was reported that, due to a typesetting mistake, the three tables and two figures for this article were included as an Additional file instead of in the body of the article. The original publication of this article has been updated to correct this.

The publisher apologises to the authors and readers for the inconvenience.
